# Compensatory gait mechanics in person with multiple toe amputation: A single case report

**DOI:** 10.1002/ccr3.7675

**Published:** 2023-08-22

**Authors:** Hirotaka Iijima, Ryo Eguchi, Yamamoto‐Kon Aya, Yuta Terabe, Masaki Takahashi

**Affiliations:** ^1^ Institute for Advanced Research Nagoya University Nagoya Japan; ^2^ Biomedical and Health Informatics Unit, Graduate School of Medicine Nagoya University Nagoya Japan; ^3^ Graduate School of Science and Technology Keio University Yokohama Japan; ^4^ Division of Fundamental Nursing, Faculty of Nursing and Medical Care Keio University Kanagawa Japan; ^5^ Kasukabe Chuo General Hospital Limb Salvage Center Saitama Japan; ^6^ Department of System Design Engineering, Faculty of Science and Technology Keio University Yokohama Japan

**Keywords:** biomechanical analysis, care report, diabetic neuropathy, toe amputation

## Abstract

This case highlights the biomechanical influence of toe amputation on contralateral limb force elevation, possibly through reduced ipsilateral plantar flexor torque production. These findings provide insight into toe amputation‐related compensatory gait mechanics with greater inter‐limb asymmetry, which may increase the risk of musculoskeletal comorbidities, including osteoarthritis in contralateral limb.

## Graphical abstract

An elderly with diabetic neuropathy and multiple toe amputation primarily in left(ipsilateral) foot. Sensor‐mounted insole‐based kinetic analyses identified a lacked push‐off in the ipsilateral limb and subsequent elevated 1st peak ground reaction force (GRF) in contralateral limb.

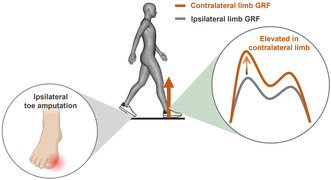

## 1 INTRODUCTION

Diabetic neuropathy and the resulting partial foot amputation cause gait alterations, including slower gait velocity, greater step variability, greater inter‐limb asymmetry, and altered ground reaction force (GRF).^1^ These biomechanical alterations with impaired walking capacity increase the risk of falls as well as musculoskeletal comorbidities, including osteoarthritis in the contralateral limb,^2^ leading to loss of functional independence.

As partial foot amputation‐induced gait alterations are prominent in proximal amputation due, at least in part, to loss of moment arm,^3^ surgical techniques have been proposed to save the foot and prolong the bipedal ambulatory function. However, the currently available data regarding partial toe amputation patients are limited. For example, patients who have undergone first ray amputation display slower gait velocity, reduced step length, greater step variability, and decreased hip extension.^4^ To date, no study has investigated the influence of non‐first ray toe amputation on gait alterations in patients with diabetic neuropathy.

Herein, we report the case of a patient who had undergone non‐first ray but multiple toe amputation, in who integrated gait analysis was performed to assess spatiotemporal parameters, kinetics, and kinematics. We demonstrated that multiple toe amputation caused a lack of push‐off with decreased hip extension in the ipsilateral limb accompanied by elevated contralateral GRF. This single case study provides insight into toe amputation‐related biomechanical alterations and serves as a framework for future studies to establish an effective rehabilitative strategy and prevent musculoskeletal comorbidities following amputation. The patient was informed that data concerning the case would be submitted for publication, and he consented to it.

## 2 CASE PRESENTATION

### 2.1 Patient information

The patient was a 65‐year‐old Japanese man with diabetic neuropathy, who had undergone multiple toe amputation (second, third, and fourth phalanxes and at least part of the metatarsal) in left limb in May 2013 (when he was 60‐year‐old) and single toe amputation (third proximal phalanx) 3 year later, May 2016. The patient had comorbidities of critical limb ischemia and received hemodialysis. Although diabetic‐related foot deformity, including Charcot foot, was not confirmed, left foot displayed clow toe after the surgery. Joint mobility in both forefoot and hindfoot was limited based on physical assessment by experienced medical doctor. He could walk independently on flat surfaces without using an ambulatory assistive device and foot orthoses, although he felt difficulty in walking. Given the multiple toe amputations in the left foot, henceforth we have used the term “ipsilateral” for the left limb and “contralateral” for the right limb.

### 2.2 Clinical findings

**FIGURE 1:**
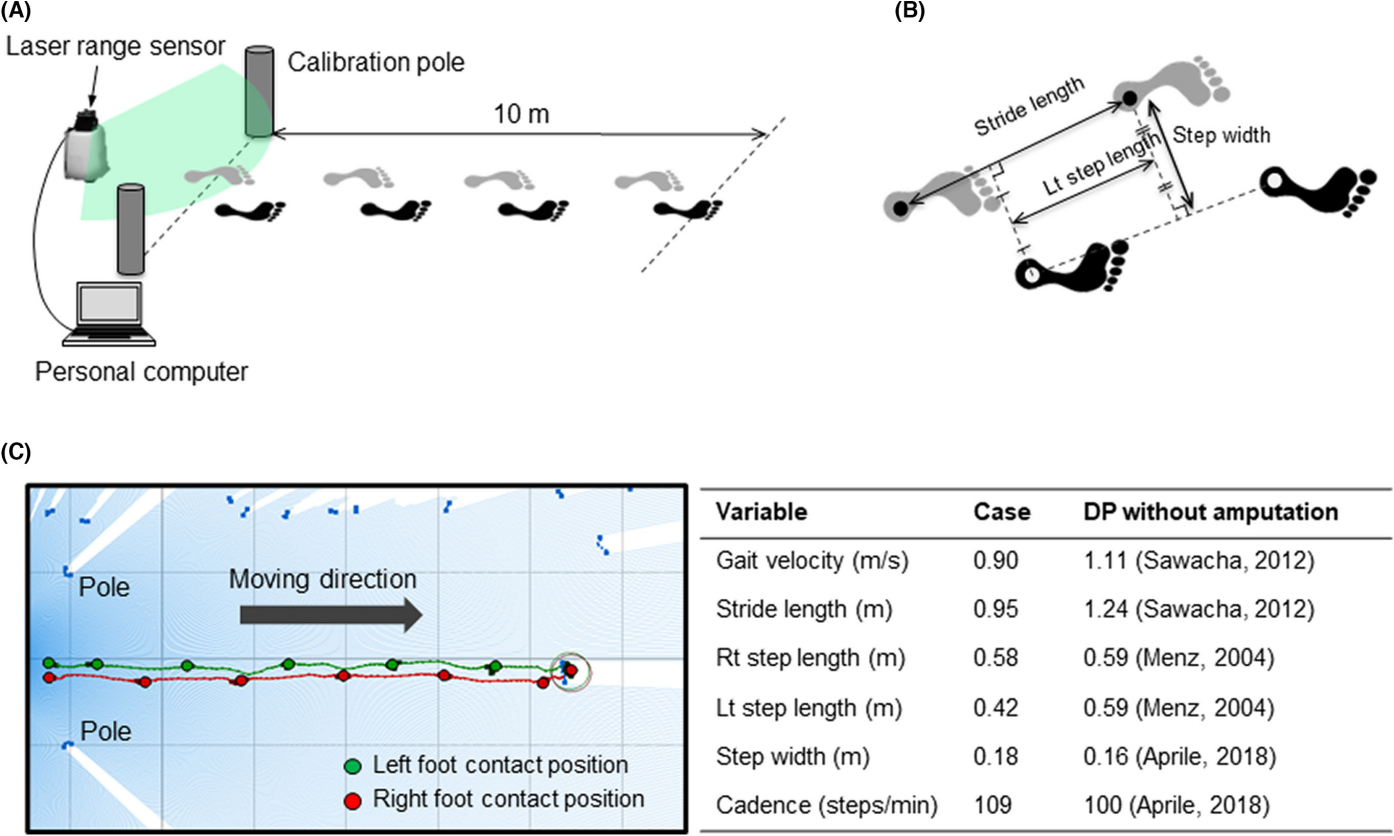
Laser range sensor‐based leg tracking system reveals poor walking capacity in the patient who have undergone toe amputation. The patient's gait being assessed in a comfortable walking style/at a comfortable walking pace using a laser range sensor (A). Calculation of spatiotemporal gait parameters (B). Results of the laser range sensor‐based gait analysis (C). Data of spatiotemporal parameters in diabetic patients (DP) without amputation are provided as a reference.^4^, ^9^, ^10^

As his primary concern was having difficulty in walking, we first sought to determine whether the patient displayed a decline in walking capacity, we evaluated spatiotemporal gait parameters during level walking using a laser range sensor‐based leg tracking system (Figure 1A).^5^ This new technology‐based gait assessment has been applied to characterize ambulation in patients with neurodegenerative and musculoskeletal diseases.^6^, ^7^, ^8^ As this analysis system evaluates gait parameters in the right and left limbs separately (Figure 1B), it provides insight into amputation‐related gait alterations. As expected, this patient displayed poor walking capacity compared to the reference value derived from patients with diabetic neuropathy without amputation,^4^, ^9^, ^10^ as evidenced by slower gait velocity, shorter stride length, and shorter step length, especially the ipsilateral step length (Figure 1C). These data indicate that patients who have undergone multiple toe amputation display gait disorders with inter‐limb asymmetry in step length.

**FIGURE 2:**
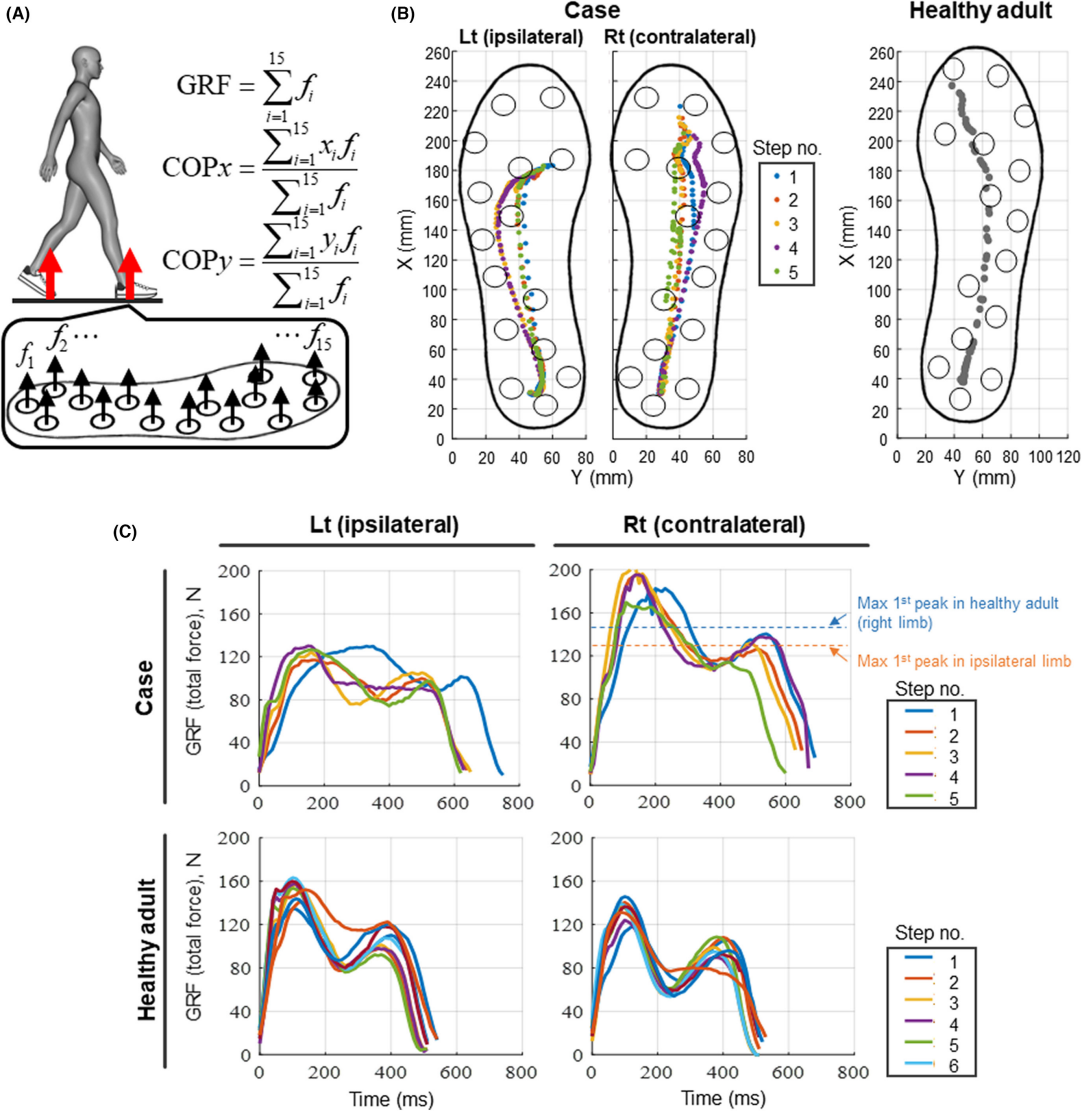
Insole‐based biomechanical analysis revealed a lack of push‐off in the ipsilateral limb and subsequent increased first ground reaction force in the contralateral limb. The sensor‐mounted insole system estimates the center of pressure (COP) and vertical ground reaction force (GRF) during level walking (A). The COP analysis in the patient revealed reduced excursion in the left (ipsilateral) limb, which was derived from a lack of push‐off motion by the forefoot (B). The COP in healthy adults is provided as a reference. The patient displayed reduced first and second peak GRF in the ipsilateral limb and increased first peak GRF compared to healthy adults (C).

As partial foot amputation influences the center of pressure (COP) and GRF,^1^ we determined whether toe amputation influences these kinematic changes. We used a validated custom‐made sensor‐mounted insole to assess the COP and GRF (Figure 2A).^11^ This system can measure a force similar to the vertical GRF by summing the forces of 15 sensors attached to the insole. The system can also estimate the COP positions in the anteroposterior and mediolateral directions by calculating the weighted average of the sensor positions and forces. We found that the ipsilateral foot displayed reduced COP excursion, which was derived from a lack of push‐off motion by the forefoot (Figure 2B). In addition, the COP during the terminal stance in the ipsilateral foot was localized at the first metatarsal, which was not observed in the COP of the contralateral foot and in healthy adults (Figure 2B). Of note, contralateral foot also displayed reduced COP excursion compared to healthy adult, suggesting that foot function in contralateral limb is not necessarily normal. The confirmed inter‐limb difference in the COP was consistent with the GRF data showing inter‐limb asymmetry (Figure 2C). On comparison with healthy adults, our patient displayed lower first and second peak values of GRF in the ipsilateral limb (Figure 2C). Notably, the first peak GRF in the contralateral limb was higher than that in healthy adults. These findings indicate that the lack of push‐off in the ipsilateral limb is related to increased first peak GRF in the contralateral limb.

**FIGURE 3:**
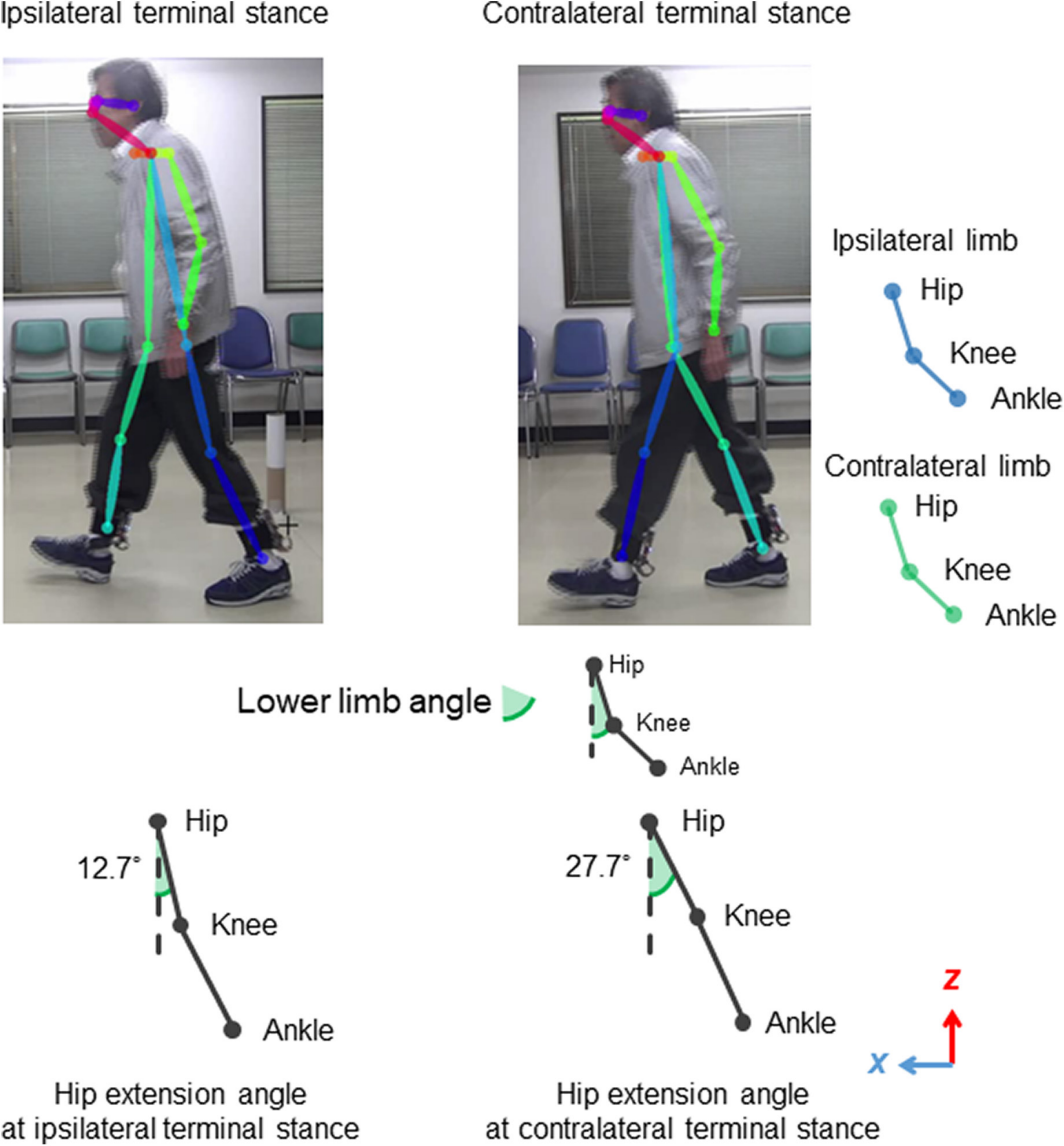
Video‐based kinematic analysis revealed reduce hip extension in the ipsilateral limb. The whole‐body skeleton was visualized during level walking using the OpenPose system. Graphic illustrations represent the terminal stance phase of the ipsilateral (left panel) and contralateral (right panel) limbs. Terminal stance in the ipsilateral limb is characterized by reduced hip extension, indicated by a lower hip extension angle (angle between the femur and the vertical line) manually measured by ImageJ software. The values of the hip joint angle are provided based on a camera coordinate system.

To further support the biomechanical inter‐limb asymmetry, we recorded level walking using a 60‐fps stationary camera (HDR‐CX550V; Sony Corp, Sony Marketing Inc.) and performed kinematic analysis based on the whole‐body skeleton visualized using the OpenPose system.^12^ We focused on the gait cycle from the ipsilateral terminal stance (i.e., contralateral heel contact), which corresponds to the observed alterations in the COP and GRF. This decision was made based on previous biomechanical evidence that the drop‐off effect during the terminal stance in one limb leads to increased GRF in the contralateral limb.^3^ Figure 3 shows the results of the gait movie analysis in the sagittal plane. The hip extension angle during the terminal stance in the ipsilateral limb was lower than that in the contralateral limb (12.7° vs. 27.7°, as measured by ImageJ software).

## 3 DISCUSSION

This single case study identified alterations in spatiotemporal gait parameters and biomechanics associated with multiple toe amputation during level walking. Most notably, the patient displayed a lack of push‐off movement with reduced hip extension during the terminal stance phase in the ipsilateral limb, which was accompanied by an elevated first peak GRF in the contralateral limb.

### 3.1 Interpretation of the observed biomechanical alterations

Comprehensive gait analysis is a fundamental step in identifying gait disorders associated with amputation. However, to date, there is limited evidence regarding biomechanical alterations in patients who have undergone toe amputation. One study showed that patients who had undergone first ray amputation displayed poor walking capacity with kinematic alterations in hip extension.^4^ The current study expands on this previous knowledge by showing that patients with a history of multiple non‐first ray amputations displayed biomechanical alterations characterized by a lack of push‐off accompanied by kinematic changes in lower hip extension. These biomechanical alterations would be attributed, at least in part, to the loss of function in the second to fourth toes. Toe amputation reduces leverage, which is required to produce plantar flexor torque during the terminal stance. It should be noted that the patient included in this study had undergone amputation of the third toe in the contralateral limb, suggesting that the poor walking capacity and biomechanical alterations might be a consequence of bilateral toe amputation.

Notably, the biomechanical alterations in the ipsilateral limb were accompanied by an increased vertical first peak GRF in the contralateral limb. The identification of biomechanical alterations in the contralateral limb is valuable, as this may provide insight into musculoskeletal comorbidities after amputation. Increased axial lower limb loading at heel contact may lead to faster progression of existing osteoarthritis.^13^ Knee osteoarthritis is a major comorbidity associated with amputation.^14^ Although no data are available on the prevalence of these comorbidities after partial toe amputation, our findings serve as a foundation for future cohort studies on musculoskeletal comorbidities after partial toe amputation.

Considering that, nearly one‐third of the individuals require re‐amputation within 1 year following an initial toe amputation,^15^ an enhanced understanding of the disease trajectory after initial amputation is needed. Elevated plantar pressure is a major risk factor of diabetic ulcers especially at metatarsal,^16^ a precursor for amputation.^17^ Our patient displayed reduced step length in the ipsilateral limb, which reduces peak plantar pressure during level walking.^18^ On the other hand, the COP during the terminal stance in the ipsilateral foot was localized at the first metatarsal, which may cause excessive plantar pressure in this region, the common location for foot ulcers.^19^ These altered biomechanics in the ipsilateral foot highlight the importance of careful observation following the initial amputation to prevent secondary amputation.

### 3.2 Practical and research implications

This case report has the following implications: (1) the lack of push‐off after toe amputation should be carefully observed, as this may cause increased contralateral limb loading and subsequent musculoskeletal comorbidities. (2) Understanding the musculoskeletal comorbidities in the contralateral limb after toe amputation is an interesting future direction to determine the relationship between the former and increased contralateral limb loading. In the future studies, careful consideration for confounders, including preexisting arthritis in multiple joints and cardiovascular status is warranted. As we cannot rule out that biomechanical changes in ipsilateral limb are consequence of those in contralateral limb, determining the causal relationship is also needed. Once the causal relationship of the lacked push‐off in ipsilateral limb and mechanical overloading with subsequent comorbidities in contralateral limb is verified, rehabilitation and gait retraining are of interest to improve the production capacity of plantar flexor torque during the ipsilateral terminal stance.^20^

## AUTHOR CONTRIBUTIONS

**Hirotaka Iijima:** Conceptualization; data curation; formal analysis; funding acquisition; investigation; methodology; project administration; resources; software; supervision; validation; visualization; writing – original draft; writing – review and editing. **Ryo Eguchi:** Data curation; formal analysis; investigation; methodology; resources; software. **Aya Yamamoto‐Kon:** Conceptualization; funding acquisition; investigation; supervision. **Yuta Terabe:** Investigation; project administration; supervision. **Masaki Takahashi:** Conceptualization; investigation; methodology; validation.

## FUNDING INFORMATION

This study was supported in part by (1) a JSPS KAKENHI (Grant Number: 23H03308) from the Japan Society for the Promotion of Science (https://www.jsps.go.jp/) for HI (2) a JSPS KAKENHI (Grant Number: 18K10363) from the Japan Society for the Promotion of Science for AYK.

## CONFLICT OF INTEREST STATEMENT

The authors declared that they have no competing interests.

## CONSENT

Written informed consent was obtained from the patient to publish this report in accordance with the journal's patient consent policy.

## Data Availability

All data generated or analyzed during this study are included in this published article.
